# Transcriptome Sequencing Investigated the Tumor-Related Factors Changes After *T. gondii* Infection

**DOI:** 10.3389/fmicb.2019.00181

**Published:** 2019-02-07

**Authors:** Gang Lu, Jian Zhou, Ying hui Zhao, Qiao ling Li, Yun yun Gao, Lin Wang

**Affiliations:** ^1^Institute of Pathogen Biology, Taishan Medical College, Tai’an, China; ^2^Department of Orthopedics, The Second Xiangya Hospital, Central South University, Changsha, China; ^3^Department of Sports Medicine Research Center, Central South University, Changsha, China; ^4^Department of Ji Nan Children’s Hospital, Jinan, China

**Keywords:** *Toxoplasma gondii*, RNA-Seq, tumor-related factors, changes, suppression

## Abstract

*Toxoplasma gondii* is an intracellular parasite and causes a global epidemic parasitic disease. *T. gondii-*infection could inhibit the growth of tumor. In this study, the transcriptomes of samples were detected by deep sequencing analysis. The transcriptome data was compared with reference genome to perform sequence alignment and the further analysis. The analyses of differential expression and the differentially expressed genes were performed in the present study. Genes involved in *P53* signaling pathway, COLORECTAL cancer pathway, NON-SMALL CELL LUNG cancer signaling pathway, and BREAST cancer signaling pathway were up-regulated or down-regulated among the samples. The KEGG analysis indicated that the cancer pathways changed after infection of *T. gondii.* Furthermore, tumor-related mRNAs from different samples had a large difference, which suggested that the difference might provide important information in resisting cancer. The protein results indicated that tumor-related protein changes occurred after infection of *T. gondii.* In conclusion, the infection changed the cancer pathways, which could possibly inhibit the growth of tumor.

## Introduction

*Toxoplasma gondii* is a kind of obligate intracellular parasite protozoan that is distributed worldwide. Most of *T. gondii* infections are latent infections and do not show clinical symptoms. However, once the body’s immune function is impaired, *T. gondii* can cause opportunistic diseases (Martins-Duarte et al., 2010). The occurrence of tumors activates the reproduction of *T. gondii* in hosts. Tumor patients are more likely to suffer toxoplasmosis than non-tumor people (Jiang et al., 2015; Al-Mendalawi, 2017; Imam et al., 2017; Vittecoq and Thomas, 2017). Conversely, *T. gondii* infection can also promote tumorigenesis? The answer is no. Recent studies have indicated that *T. gondii* infection can’t cause tumors, but can inhibit the occurrence of tumors (Baird et al., 2013; Fox et al., 2013; Sanders et al., 2015, 2016; Pyo et al., 2016; Fox et al., 2017). Uracil-auxotrophic *T. gondii* confers long-term effective immunity to diffuse pancreatic cancer. *T. gondii* enhances the response of host anti-tumor CD8+ T cells and prolongs the survival time of tumor-inoculated mice (Sanders et al., 2015, 2016). Furthermore, uracil-auxotrophic *T. gondii* can preferentially invade tumor-associated antigen-presenting cells and can stimulate the anti-ovarian tumor response of anti-tumor CD8+ T cells (Baird et al., 2013).

Profilin-like protein (TgPLP) of *T. gondii* is a host toll-like receptor (TLR) activator that activates TLR11 and dendritic cells (DCs) (Yarovinsky et al., 2005; Hatai et al., 2016). Innate recognition plays an important role in immune response of host (Plattner et al., 2008; Mizel and Bates, 2010; Koblansky et al., 2013). The research indicates that TgPLP can enhance the immune response of autologous whole-tumor-cell vaccine (AWV). Similarly, *T. gondii* virulence-associated molecule of dense granule protein (ToxoGRA15II) can induce macrophage polarization to M1, which has a limiting effect on tumor growth (Li et al., 2017). In addition, rhoptry protein 5, 17, 18, 35, and 38 (ROP5, ROP17, ROP18, ROP35, and ROP38), dense granule protein 2, 12, and 24 (GRA2, GRA12, and GRA24) can induce the anti-tumor immune response (Fox et al., 2016). Although these molecules play a role in stimulating immunity in anti-tumor, the mechanism remains unclear. Is there any tumor-related factor participate in the response? So it is necessary to analyze the products of tumor-related factors before and after infection with *T. gondii* in mice.

## Materials and Methods

### Mice and Parasites

Eight-week-old female BALB/c mice were obtained from Shandong University Laboratory Animal Center. They were bred in groups of twelve per cage under specific-pathogen-free conditions and were free to diet and tap water. They were bred in 12 h of continuous lighting every day at 25°C. *T. gondii* (low virulent PRU strain) was maintained from our laboratory using passage of cysts in Kunming mice. The treated mice were challenged intragastrically with 20 cysts of *T. gondii* PRU strain. The mice without cysts-challenge were used as control group. All of the animal experiments were approved by the Ethics Committee on Animal Experiments of the Medical School of Shandong University. BALB/c mice were challenged with cysts and the spleens were collected for mRNA extraction after a month.

### Total mRNA Extraction

The total mRNA of cell extracted used TRIZOL for isolation of total RNA according to the previous protocol. Smartspecplus (BioRad) was used to measure the absorption value 260/280 nm (A260/A280) to qualify and quantify the collected RNA. Lastly, the integrity of the extracted RNA was further detected using 1.5% agarose gel electrophoresis. Subsequently, the RNA was transcribed to first strand cDNA by the First Strand cDNA Synthesis Kit (TAKARA) for gene expression analysis.

### RNA-Seq Library Construction and Sequencing

A total of 3 μg RNA per sample was used as input material for the RNA sample preparations. Sequencing libraries were generated using NEBNext^®^Ultra^TM^ RNA Library Prep Kit for Illumina^®^ (NEB, United States), which were added to attribute sequences to each sample. Briefly, mRNA was purified from total RNA using poly-T oligo-attached magnetic beads. Fragmentation was carried out using divalent cations under elevated temperature in NEBNext First Strand Synthesis Reaction Buffer (5×. First strand cDNA was synthesized using random hexamer primers and M-MuLV Reverse Transcriptase (RNase H-). Second strand cDNA synthesis was subsequently performed using DNA Polymerase I and RNase H. Remaining overhangs were converted into blunt ends via exonuclease/polymerase activities. After adenylating the 3’ends of DNA fragments, NEBNext Adaptor with hairpin loop structure was ligated to prepare for hybridization. In order to preferentially select cDNA fragments ∼150-200 bp in length, the library fragments were purified with AMPure XP system (Beckman Coulter, Beverly, United States). Subssequently, 3 μl USER Enzyme (NEB, United States) was used with size-selected, adaptor-ligated cDNA at 37°C for 15 min followed by 5 min at 95°C before PCR. Then, PCR was performed with Phusion High-Fidelity DNA polymerase, Universal PCR primers and Index (X) Primer. Finally, PCR products were purified (AMPure XP system) and library quality was assessed with the Agilent Bioanalyzer 2100 system.

According to Sequencing by Synthesis (SBS) technology, the Illumina HiSeq2500 high-throughput sequencing platform was used to sequence the cDNA library and it obtained significant amounts of high-quality reads. The reads and the sequenced-bases were usually considered to be Raw Data and most of them got a Q30 score for base quality.

### Annotation and Analysis of the Differentially Expressed Genes

On the basis of method described by Audic and Claverie, we defined the cut-off values to collect the differentially expressed genes. In order to quantify expression, the coverage from every transcript was analyzed with applying BEDtools v. 2.9.0 (Quinlan and Hall, 2010). The coverage of all libraries was then integrated into a single file. The file was predicted using R v. 3.0.1 with the bioconductor package ‘DEGseq’ v. 1.2 (Wang et al., 2010). A proven Benjamini-Hochberg correction method was used to correct the significant *p*-value in the analysis of differential expression, and then FDR was used as the key indicator of screening of the differentially expressed genes.

GO database is a standard structured biological annotation system, aimed at establishing a system of standard vocabulary and knowledge of genes and their products. The GO annotation system contains three main branches: Biological Process, Molecular Function, and Cellular Component. GO database was used to predict the functions of the differentially expressed genes in the study. Enrichment analysis of the differences between samples was carried out using Top GO. The cluster of orthologs groups of proteins (COG) database is constructed on the basis of phylogeny of bacteria, algae and eukaryotes to classify the orthologs of the gene product. To annotate the pathway of the differentially expressed genes, it is necessary to further analyze the function of the genes. The kyoto encyclopedia of genes and genomes (KEGG) database is a major metabolic pathways database. In the present study, COG and KEGG were used to analyze the differentially expressed genes.

### Real-Time PCR Analysis

Total RNA was extracted from independent biological replicates using RNA extraction kit on the basis of the manufacturer’s protocol. 10 μg of total RNA was subjected to DNaseI treatment with 1 U DNaseI (NEB, United States). The reaction was carried out at 37°C for 10 min followed by heat inactivation at 65°C for10 min. 2.5 μg of DNase-treated RNA was used for cDNA synthesis with reverse transcriptase (BioRad, United States) according to the manufacturer’s protocol. The expression of *Gapdh* gene was detected to be stable in the transcriptome database, and it was used as the control in qRT-PCR. Primers were designed for selected transcripts from the transcriptome database and real time PCR was performed by SYBR green I master mix (Roche, GmBH) on CFX-ConnectTM Real time system (BioRad, United States). Relative expression of the transcripts was calculated by the ΔΔCt method.

### Western Blot Analysis

Western blot analysis of spleen cells was performed as below: The cells were treated using RIPA Lyses Buffer (50mM Tris pH 7.4, 150mM NaCl, 1% Triton X-100, 1% Sodium deoxycholate, 0.1% SDS) containing 1mM protease inhibitor PMSF (phenylmethanesulfonyl fluoride) and centrifuged at 13,000 ×*g* for 10 min. Then supernatant was extracted and resuspended in 50 μl of SDS-PAGE sample buffer, and boiled for 5 min and 20 μl was loaded onto corresponding polyacrylamide gel. Proteins were transferred onto PVDF (polyvinylidene fluoride) membrane via electrophoresis, carried at 80 V for 3 h, using Bio-Rad transfer system (Bio-Rad, Hercules, CA, United States). The membrane was saturated for 2 h with sealing fluid at room temperature and probed with corresponding antibody (Goat) diluted 1:10,000 in saturation buffer. The membrane was incubated for 2 h with a HRP (horseradish peroxides)-labeledrabbit anti-goat IgG antibody (Sigma, United States) diluted 1:20,000 in saturation buffer, and signals were detected with super sensitive signal ECL (Enhanced Chemiluminescence) system.

### Statistical Analysis

Data are presented as mean ± SD. We performed statistical analyses with ANOVA or the unpaired two tailed Student’s *t*-test. All statistical analyses were performed using SPSS 17.0 software, and values of *P* < 0.05 were considered statistically significant.

## Results

### mRNA Transcriptome Library Construction, Sequencing, and Sequence Analysis From Infected Mice

The sequencing results show that a total of 62,882,826 raw reads for the control group and 58,159,644 raw reads for treated group (Table 1), a total of 55,312,920 mapped reads for the control group and 48,449,292 mapped reads for treated group (Table 1) were detected. In control group, the numbers of unique mapped reads and multiple mapped reads were 7,569,906 and 2,690,548 separately, which make up 12 and 4.3% of the raw reads. A total of 9,710,352 unique mapped reads and 3,043,845 multiple mapped reads were selected from treated group in this study.

**Table 1:** The distributions of mapped reads from T01 and T02.

	T01		T02	
		Percentage		Percentage
Statistical content	Number	(%)	Number	(%)
Total reads	62882826	100	58159644	100
Mapped Reads	55312920	87.96	48449292	83.30
Unique mapped reads	7569906	12	9710352	16.7
Multiple mapped reads	2690548	4.3	3043845	6.28

*T01, control group; T02, *T. gondii* challenged group*.

### Differential Expression of Genes

**FIGURE 1:**
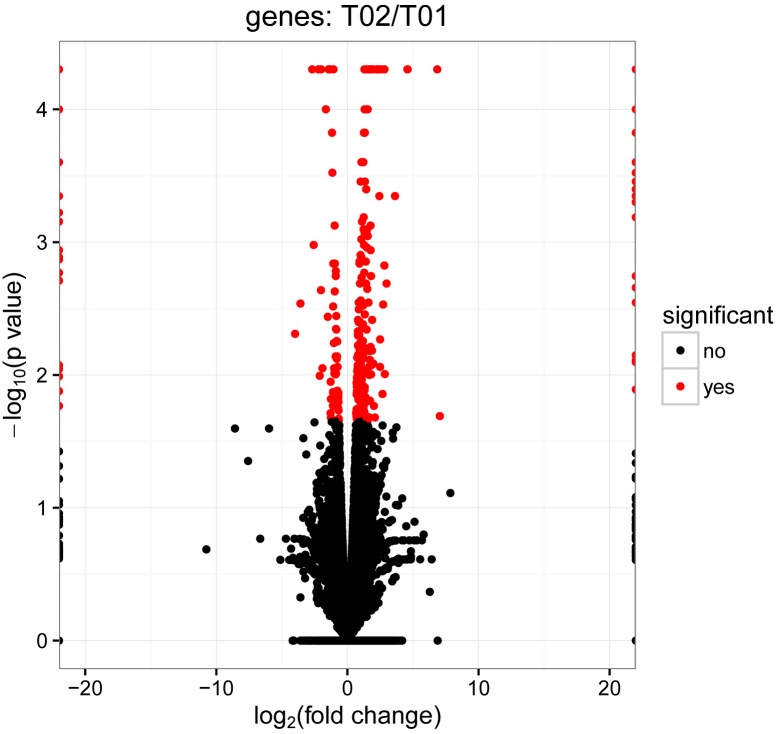
Volcano plots of differentially expressed genes between control group and infected group. In the volcano plots, each dot is a gene, the “red” genes are those that were significantly regulated in infected group compared with control group at a *q*-value < 0.05. However, the “black” genes shown there were no significantly difference between control group and infected group. In total, 432 unigenes were identified as differentially expressed, including 307 genes that were up-regulated and 125 genes that were down regulated.

**FIGURE 2:**
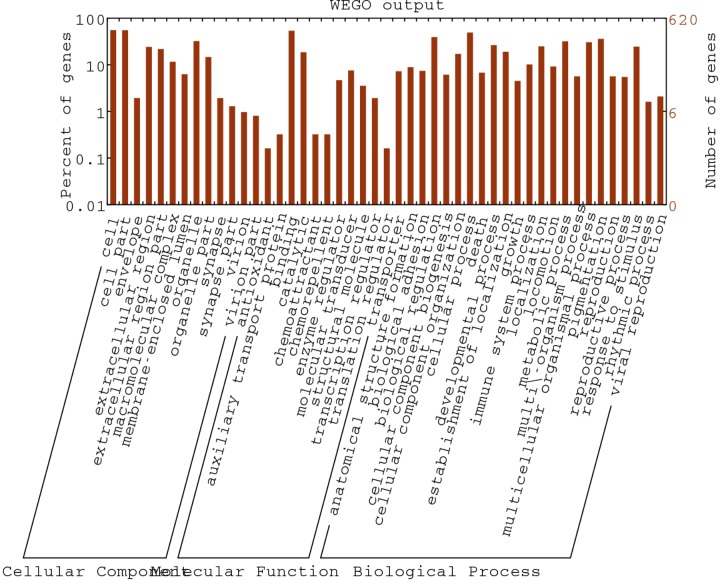
Gene Ontology classification of assembled unigenes. The DEGs genes were classified into 3 functional categories: molecular function, biological process, and cellular component. The y-axis indicates the number of genes in a category. The x-axis indicates the specific category of genes in that main category.

**FIGURE 3:**
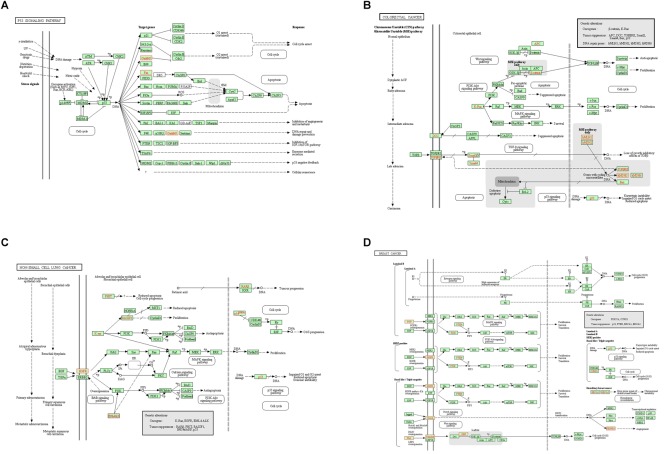
Unigenes predicted to be involved in the p53 signaling pathway **(A)**, colorectal cancer pathway **(B)**, non-small cell lung cancer pathway **(C)**, and breast cancer pathway **(D)**. Red indicates significantly different expression in infected group compared with control group.

In order to deeply explore the changes of tumor-related factors, RNA-seq was performed. In the result, 432 differences expression genes were identified. Compared with the control group, 307 up regulated genes and 125 down-regulated genes were found (Figure 1). The heatmap of DEGS is shown in Appendix Figure A1. The GO defines classes used to describe gene function, and relationships between these genes. Figure 2 presented the GO analysis of DEGs in the result. The GO terms of DEGs was mainly enriched in the biology regulation, biological process, cellular component, and molecular function. The KEGG PATHWAY is a collection of manually drawn pathway maps representing our knowledge on the molecular interaction and reaction networks. In this study, the KEGG PATHWAY analysis of DEGs is shown in the Figure 3. The pathway terms enriched by DEGs were mainly in the cancer pathways, RAS signaling pathway, RAP1 signaling pathway, calcium signaling pathway, MAPK signaling pathway and Oxytocin pathway, etc. The levels of expression of *Gadd45* and *Fas* changed significantly after *T. gondii*-challenge. The levels of expression of *APC, Smad2, Smad4, Bax, hMLH1, hMSH2, hMSH3, p53, hMSH6* changed significantly after *T. gondii*-challenge in colorectal cancer pathway. Similarly, the levels of expression of some genes changed significantly after *T. gondii*-challenge in non-small cell lung cancer and breast cancer pathways (Figure 3). The results indicated that *T. gondii*-infection may influence cancer signaling pathways.

### Further Confirmation Using qRT-PCR

**FIGURE 4:**
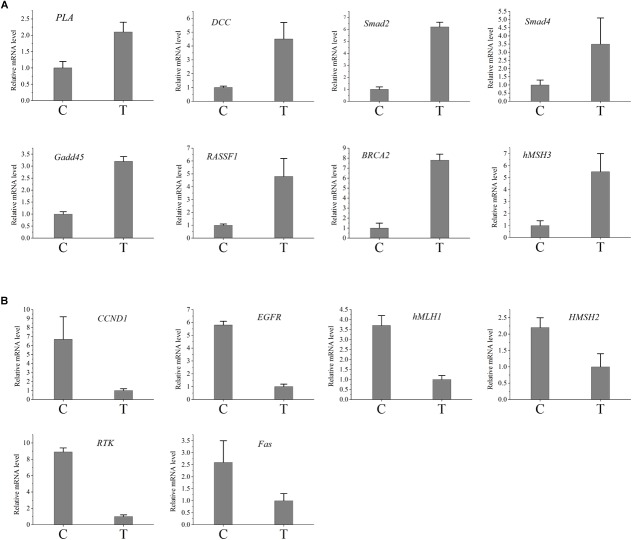
Validation of the difference in expression of the genes using qRT-PCR. **(A)** The qRT-PCR results of the up-regulated genes in infected group. **(B)** The qRT-PCR results of the down-regulated genes in infected group. C, control group; T, treated group.

In order to verify the RNA-seq results, qRT-PCR was performed in this study. The candidate genes were selected from the cancer metabolic signaling pathway. Among these genes, *CaM, RTK, PLA* genes were involved in the RAS signaling pathway; *Gadd45* and *Fas* genes belong to the *P53* signaling pathway; *APC, DCC, Smad2, Smad4, Bax, hMLH1, hMSH2, hMSH3 and hMSH6* genes were involved in the COLORECTAL cancer pathway; *K-Ras, EGFR, FHIT, RASSF1* and *RARβ* genes belong to NON-SMALL CELL LUNG cancer signaling pathway; *PTEN, BRCA1, BRCA2, PI3K, and CCND1*were involved in the BREAST cancer signaling pathway. Figure 4 presented the RT-PCR results. Compared with control group, genes *PLA, Gadd45, DCC, Smad2, Smad4,hMSH3, RASSF1* and *BRCA2* were up-regulated, while the gene *RTK, Fas, hMLH1, hMSH2, EGFR, and CCND1* were down-regulated in the treated samples. The results indicated that expressions of cancer-related genes obtained by qRT-PCR were nearly consistent with those obtained with RNA-seq.

### Changes of Tumor-Related Factors

**FIGURE 5:**
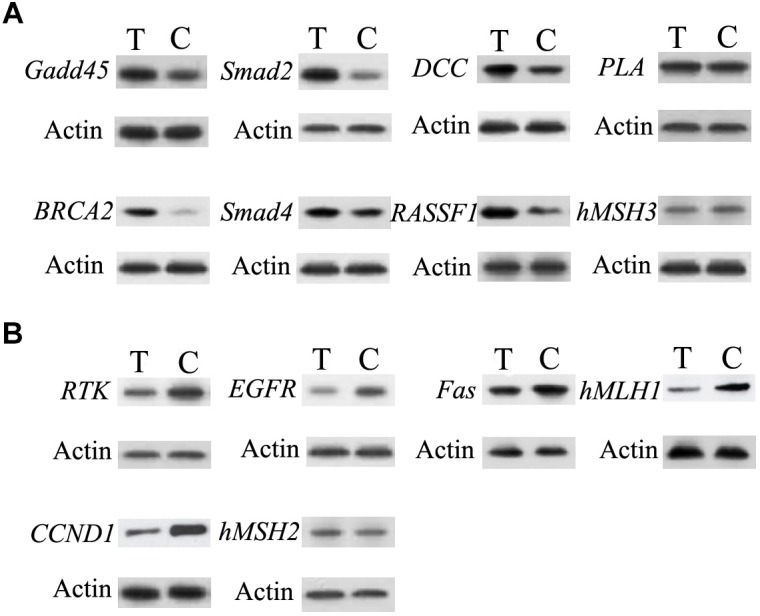
Validation of the difference in expression of the genes by western blot. **(A)** The western blot results of the up-regulated genes in infected group. **(B)** The western blot results of the down-regulated genes in infected group. C, control group; T, treated group.

Western blotting analysis of *PLA, Gadd45, DCC, Smad2, Smad4, hMSH3, RASSF1, BRCA2,RTK, Fas, hMLH1, hMSH2, EGF,R and CCND1* were performed. As shown in Figure 5, compared to control group, the levels of *DCC, Smad2, BRCA2*, and *RASSF1* proteins from treated group were higher. Moreover, the levels of *PLA, Gadd45, Smad4*, and *hMSH3* proteins of treated group were similar to those of control group. Compared to control group, the levels of *RTK, hMLH1, EGFR, and CCND1* proteins from treated group were lower. Furthermore, the levels of *Fas* and *hMSH2* proteins of treated group were similar to those of control.

## Discussion

Hosts always allow development of latent infections of *T. gondii*, which is necessary for the parasite to spread to new hosts (Denkers et al., 2012). *T. gondii* could secrete special proteins to extensively manipulate the host cells (Hunter and Sibley, 2012). Interestingly, some secreted proteins could improve the immunity against tumors in hosts (Baird et al., 2013; Fox et al., 2013, 2017; Sanders et al., 2015, 2016; Pyo et al., 2016). Previous results indicated the secreted proteins were able to resist tumors through improving the immunity of hosts. Importantly, the present result suggested *T. gondii-*infection changed the expressions of tumor-related factors, which might improve the anti-tumor ability of hosts.

The differential expression from the control and infected group had been detected using the comparative analysis of their transcriptomes. In infected sample, compared with control material, more genes were up-regulated, which suggested that infection could change the host’s genes expression. Moreover, compared to our study, more different expression genes were found in Zhou’s study (Zhou et al., 2016). Obviously, Zhou’s study had been obtained from infected pK-15 cells, while our experimental samples were from mice, which might be the reason causing different expression genes. He et al. (2016) revealed the mRNA change of mouse splenocyte organelle components after *T. gondii* infection, which only analyzed a little mRNAs from mouse spleen. In this study, we had systematically analyzed the mRNAs of mouse spleen, especially tumor-related factors.

As a classic cancer suppressor, *p53* could encode a special protein which inhibits oncogenesis by inducing senescence, apoptosis, growth arrest, and angiogenesis inhibition. Mutations in the *p53* gene (TP53) are common associated with an increased susceptibility to form cancer, and inactivation of *p53*-regulated pathways has been described in over 50% of all human cancers (Bullock and Fersht, 2001). *P53* mutation is a driver of lung cancer and inactivation is a common situation in lung cancer (Jamal-Hanjani et al., 2017). Regain activation of *p53* is favorable for lung cancer treatment (Wang and Sun, 2010). *P53* could accumulate breast cancer cells, which plays an important role in breast cancer growth (Ferraz da Costa et al., 2018). So *p53* signaling pathway plays an important role in human cancers. In the present study, *p53* signaling pathway had been changed after *T. gondii* infection. After *T. gondii* challenge, the expression of *Gadd45* protein was up-regulated, while the expression of *Fas* protein was down-regulated, which affected *p53* signaling pathway. Moreover, *Fas* protein plays an important role in suppressing colon cancer immune evasion (Xiao et al., 2018). Human surfactant protein D induces apoptosis in pancreatic cancer cell lines via *Fas*-mediated pathway (Kaur et al., 2018). *Gadd45* protein could suppress the development of leukemia (Wiest, 2018), while up-regulation of GADD45 induced cell cycle arrest and apoptosis in colorectal cancer cells (Huang et al., 2017). Therefore, *Fas* down-regulation and *Gadd45* up-regulation in the treated mice may change the resistance for cancer.

In our study, the expressions of *DCC, Smad2, Smad4, hMLH1, hMSH2*, and *hMSH3* proteins had been changed, which regulated the colorectal cancer pathway. As one of genetic marker for diagnosis of colorectal cancer, *DCC* could inhibit the growth of colorectal cancer (Hao et al., 2017). The expression of *DCC* protein was up-regulated after *T. gondii* infection, which suggested that *T. gondii* infection might improve the resistance of host against colorectal cancer through increasing the expression of *DCC* protein. Similarly, *Smad2 and Smad4* proteins had ability to inhibit the growth of colorectal cancer (Cai et al., 2018; Hirsch et al., 2018). Our result showed that the expression of *Smad2* (not *Smad4*) protein was up-regulated after *T. gondii* infection, which indicated the enhanced ability against colorectal cancer. In addition, *hMLH1, hMSH2*, and *hMSH3* proteins play important roles in colorectal cancer pathway. Their methylation affect survival in patients with colorectal cancer (Kim et al., 2018). The expression of *hMLH1* protein was down-regulated, while the expressions of *hMSH2* and *hMSH3* proteins had no change after infection, which further suggested the relevance between *T. gondii* infection and resistance to colorectal cancer.

The expressions of proteins from NON-SMALL CELL LUNG cancer signaling pathway had been analyzed in this study. *RASSF1*, one of NON-SMALL CELL LUNG tumor suppressors, play an important role in resisting the growth of NON-SMALL CELL LUNG cancer (Dubois et al., 2016; Wang H. et al., 2018). The expression of *RASSF1* protein was up-regulated after challenge in the present study, which indicated that *T. gondii* infection might improve the resistance for NON-SMALL CELL LUNG cancer. Conversely, *EGFR* could improve the growth of NON-SMALL CELL LUNG tumor (Saboundji et al., 2018; Wang Y. et al., 2018). Compared to control group, the expression of *EGFR* protein of challenged group was down-regulated, which suggested that *T. gondii* infection might inhibit the growth of NON-SMALL CELL LUNG tumor by reducing the expression of *EGFR* protein.

*PTEN, BRCA1, BRCA2, PI3K, and CCND1* genes were related to the BREAST cancer signaling pathway. *PTEN, BRCA1*, and *BRCA2* proteins play an important role in resisting the growth of BREAST cancer (Barnes-Kedar et al., 2018; El Botty et al., 2018; Sirisena et al., 2018), while *PI3K and CCND1* proteins could improve the growth of BREAST cancer (Ortiz et al., 2017; Sirisena et al., 2018). In this study, the expression of *BRCA2* was up-regulated, while *CCND1* protein was down-regulated, which indicated that the infection might inhibit the growth of BREAST cancer.

*BRCA2, Smad4, EGFR genes* play important role in pancreatic cancer pathway. As shown in Appendix Figure A1, the expressions of tumor suppressors (*p16, p53, BRCA2*, and *Smad4*) had been changed after *T. gondii* infection. Importantly, the expressions of *BRCA2* and *Smad4* genes were up-regulated after challenge, which suggested the infection might also inhibit the developing of pancreatic cancer of host. Sanders et al. revealed that attenuated *T. gondii* could treat disseminated pancreatic cancer through generating long-lasting immunity (Sanders et al., 2016). Given the results of the present study, *T. gondii* could not only generate immunity but also change the tumor-related factors of host. Furthermore, lots of tumor-related factors of glioma pathway, prostate cancer pathway, basal cell carcinoma pathway, melanoma pathway, bladder cancer pathway, and endometrial cancer pathway had been changed after challenge (Appendix Figure A2), suggesting the infection of *T. gondii* might widely regulate the expressions of tumor-related proteins in host.

Infection of *T. gondii* could cause many changes of physiological phenomenon in host. Previous researches verified the enhanced immunity against tumor in infected hosts. In the present study, lots of tumor-related proteins were detected and many of them had been changed after challenge. Importantly, the changes were possibly beneficial to the suppression of tumor.

## Author Contributions

LW conceived and designed the study, and critically revised the manuscript. GL carried out the experiments and drafted the manuscript. JZ, YZ, QL, and YG contributed to the revision of the manuscript. All authors read and approved the final manuscript.

## Conflict of Interest Statement

The authors declare that the research was conducted in the absence of any commercial or financial relationships that could be construed as a potential conflict of interest.

## Supplementary Material

FIGURE A1 The heat map of the differently expressed genes. T01, control group; T02, *T. gondii*-infected group.

FIGURE A2 Unigenes predicted to be involved in endometrial cancer pathway **(A)**, glioma pathway **(B)**, prostate cancer pathway **(C)**, basal cell carcinoma pathway **(D)**, melanoma pathway **(E)**, and bladder cancer pathway **(F)**. Red indicates significantly different expression in infected group compared with control group.
