# Supplementary figures and images for: Transcriptome Sequencing Investigated the Tumor-Related Factors Changes After T. gondii Infection
Source: Front Microbiol. 2019 Feb 7;10:181. doi: 10.3389/fmicb.2019.00181 (PMC6374557; doi:10.3389/fmicb.2019.00181)

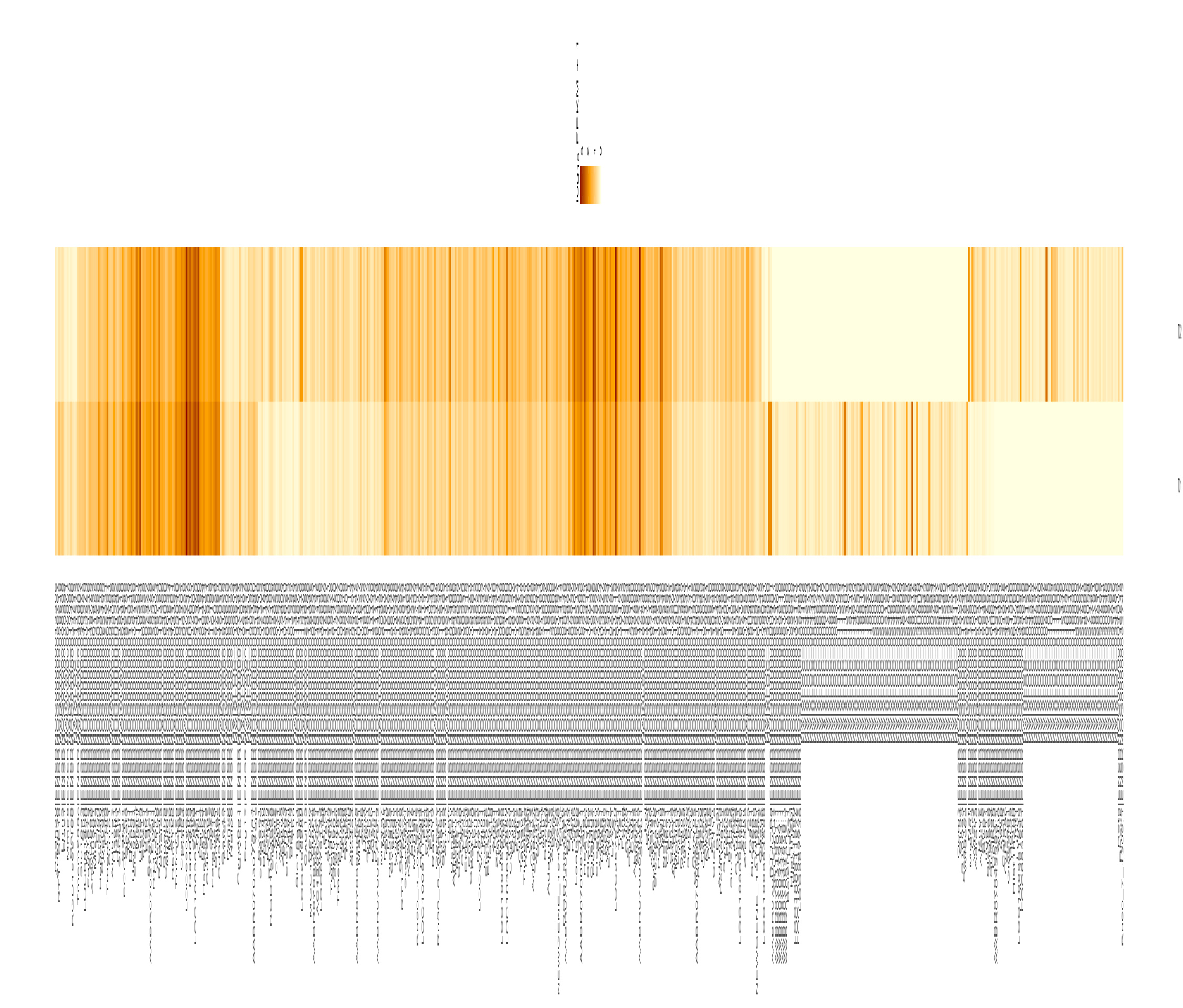

Supplement: FIGURE A1 — The heat map of the differently expressed genes. T01, control group; T02, T. gondii-infected group. [file Image_1.JPEG]

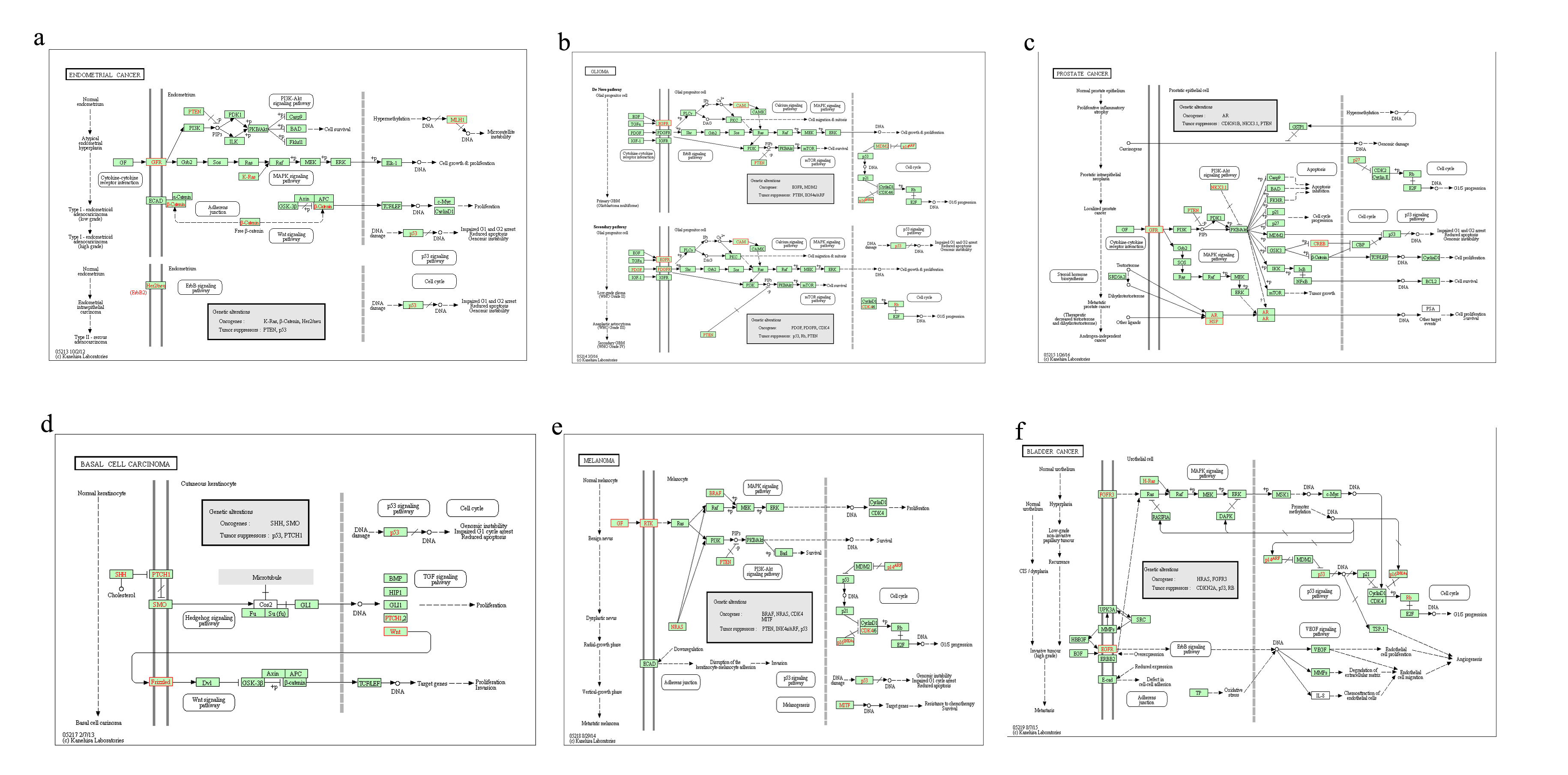

Supplement: FIGURE A2 — Unigenes predicted to be involved in endometrial cancer pathway (A), glioma pathway (B), prostate cancer pathway (C), basal cell carcinoma pathway (D), melanoma pathway (E), and bladder cancer pathway (F). Red indicates significantly different expression in infected group compared with control group. [file Image_2.JPEG]
